# Recent Progress of Wide Bandgap Perovskites towards Two-Terminal Perovskite/Silicon Tandem Solar Cells

**DOI:** 10.3390/nano14020202

**Published:** 2024-01-16

**Authors:** Qianyu Chen, Long Zhou, Jiaojiao Zhang, Dazheng Chen, Weidong Zhu, He Xi, Jincheng Zhang, Chunfu Zhang, Yue Hao

**Affiliations:** 1 National Key Laboratory of Wide Bandgap Semiconductor Devices and Integrated Technology and Shaanxi Joint Key Laboratory of Graphene, School of Microelectronics, Xidian University, Xi’an 710071, China; 2 Xi’an Baoxin Solar Technology Co., Ltd., Xi’an 710071, China

**Keywords:** wide bandgap perovskites, additive engineering, interface modification, perovskite/silicon tandem solar cells

## Abstract

Perovskite/silicon tandem solar cells have garnered considerable interest due to their potential to surpass the Shockley–Queisser limit of single-junction Si solar cells. The rapidly advanced efficiencies of perovskite/silicon tandem solar cells benefit from the significant improvements in perovskite technology. Beginning with the evolution of wide bandgap perovskite cells towards two-terminal (2T) perovskite/silicon tandem solar cells, this work concentrates on component engineering, additives, and interface modification of wide bandgap perovskite cells. Furthermore, the advancements in 2T perovskite/silicon tandem solar cells are presented, and the influence of the central interconnect layer and the Si cell on the progression of the tandem solar cells is emphasized. Finally, we discuss the challenges and obstacles associated with 2T perovskite/silicon tandem solar cells, conducting a thorough analysis and providing a prospect for their future.

## 1. Introduction

Silicon solar cells dominate the photovoltaic industry due to their high efficiency, low production costs, and high reliability. Assuming radiation losses at the front and back of the solar cell, the maximum efficiency of 33.16% is achieved at a bandgap energy of 1.34 eV (1.14 eV for silicon) [1]. However, due to the strong Auger recombination, the efficiency limit of silicon cells (≈29%) is much lower than the Shockley–Queisser efficiency limit (≈33%) [2]. At present, the power conversion efficiency (PCE) of silicon solar cells has reached 27.1%, which is relatively close to the efficiency limit and has little room for improvement [3]. Importantly, the record-certified PCE of perovskite/silicon tandem solar cells has been approved at 33%, showing a significant potential in photovoltaic fields. However, the durability and stability of perovskites further limited their application in industrialization. Even with these results, perovskite solar cells are promising to further improve efficiency and durability via changing perovskite composition, applying additive passivation, structural engineering of grain orientation, modification of the precursor solution, and innovative postprocessing [4]. In addition, PSCs are ideal candidates to achieve high power conversion efficiency and are easy to combine with other types of solar cells like silicon, copper indium gallium selenide (CIGS), narrow band gap PSCs, organic, and CdTe solar cells [5]. Among these interesting tandems, we mainly focus on the 2T perovskite/Si tandem solar cell, which achieves the highest record with a PCE of 33.9% [6]. The top cells with a wide bandgap can absorb high-energy photons with lower thermalization losses. In contrast, the bottom cells with a narrow bandgap made of c-Si effectively absorb the low-energy photons from the solar spectrum [7]. Perovskites are strong contenders for top cells in tandem architecture with silicon due to their low Urbach energy, tunable band gap (E_g_), and high absorption coefficient. The low Urbach energy provides high open circuit voltage (V_oc_) and a remarkably high tolerance to crystalline defects [8,9]. Fortunately, the affordable cost and abundance of materials suggest that perovskites could be suitable for terawatt-scale mass production if sufficient durability can be achieved [10].

**Figure 1:**
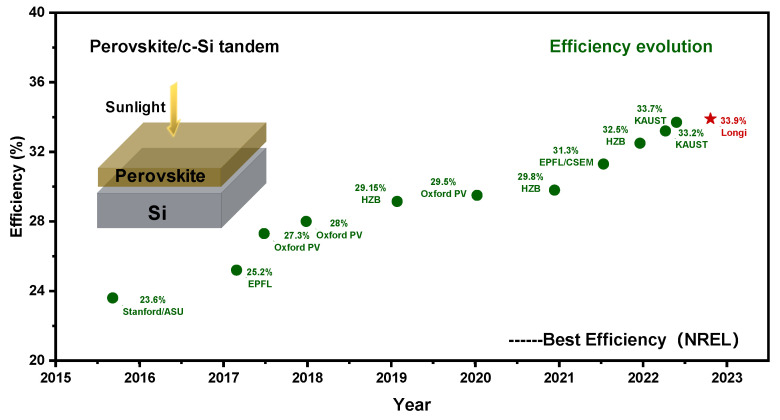
Efficiency evolution of perovskite/silicon tandem solar cells. These values come from the National Renewable Energy Laboratory’s certification.

With the rapid development of perovskite solar cells, the power conversion efficacy of perovskite/silicon tandem solar cells has risen from the original 13.7% [11] to 33.9% [12]. The highest efficiency has been certified by the National Renewable Energy Laboratory (NREL) and is achieved by the Chinese photovoltaic company Longi Green Energy, as shown in Figure 1. This momentous accomplishment demonstrates that tandem solar cells can exceed the efficiency restrictions of single-junction silicon solar cells. Consequently, perovskite/silicon tandem solar cells have the potential to enhance solar energy efficiency and diminish solar energy expenses.

**Figure 2:**
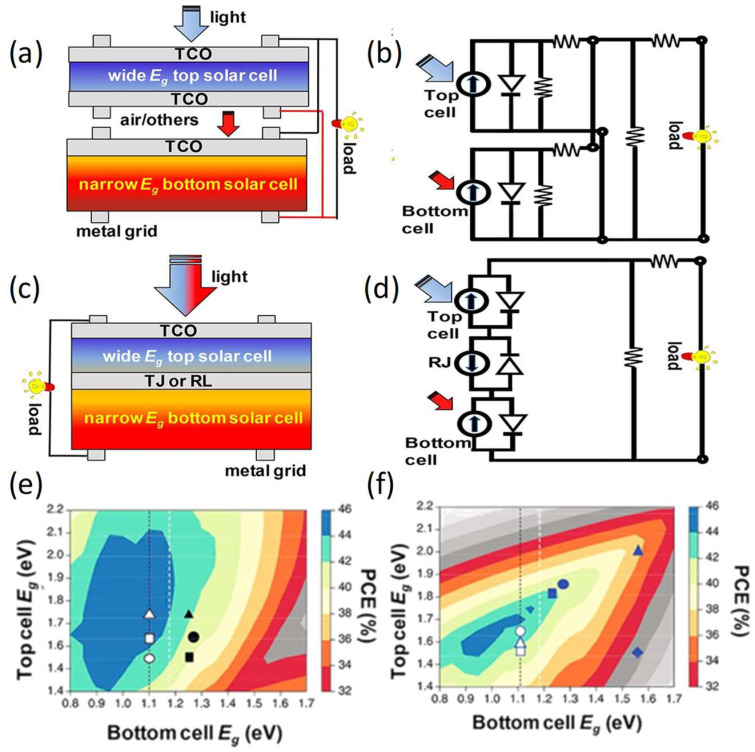
Schematic diagrams and equivalent circuits of (**a**,**b**) 4T tandem solar cells and (**c**,**d**) 2T tandem solar cells [14]. Copyright © 2020, American Chemical Society. Theoretical PCEs for (**e**) 4T and (**f**) 2T TSCs. The dotted white lines mark the lowest bandgap currently accessible to metal halide perovskite semiconductors. The black dashed line marks the 1.12 eV bandgap of silicon. The solid symbols represent the bandgap combinations currently used to fabricate the full perovskite series, and the open symbols represent the bandgap combinations realized for the 2T perovskite-silicon series (details in ref. [15]). Copyright © 2018, Springer Nature.

Perovskite/silicon tandem solar cells can be divided into two-terminal structures and four-terminal structures according to their structure (Figure 2a–d). In the four-terminal perovskite/silicon tandem solar cell, the two subcells are manufactured independently and mechanically stacked. Since each subunit can be manufactured or optimized independently, it has the advantage of unconstrained current matching [13]. Thus, the band gap of perovskites for the 4T perovskite/silicon tandem solar cell exhibited a large range from 1.4 to 2.1 eV, as shown in Figure 2e. However, the intricate fabrication and the increased material costs produced by the multiple substrates typically lead to heightened overall costs. The two-terminal (or monolithic) perovskite/silicon tandem solar cells comprise a wide bandgap cell and a narrow bandgap silicon cell. It contains transparent front and opaque rear electrodes, with the front and rear units connected through an interconnect layer (ICL). The primary benefit of the two-terminal tandem configuration is its simplicity, which may result in decreased manufacturing complexity and potentially lower costs. Additionally, the 2T tandem configuration is directly fabricated on Si substrate, making it more advantageous regarding light collection than the four-terminal tandem configuration. Nevertheless, a challenge for the 2T tandem is the current alignment between the perovskite and silicon subcells to minimize power dissipation. Therefore, wide bandgap perovskites (1.68–1.8 eV) are used to achieve better current alignment (Figure 2f). Meanwhile, optimizing the perovskite top cell, the intermediate interconnect layer, and the crystalline silicon bottom cell are essential to enhancing the efficiency of tandem solar cells.

Herein, we summarized the recent progress of wide bandgap perovskite solar cells towards 2T perovskite/silicon tandem solar cells. We systematically discussed the components engineering, additives engineering, and interface engineering of wide bandgap perovskite cells. Furthermore, we provided a prospect to achieve high efficiency for perovskite/silicon tandem solar cells.

## 2. Compositional Engineering

The bandgap and film stability of perovskites could be modified with compositional engineering. Though bandgaps may be similar, diverse perovskite elements can form various crystal and electronic structures, ultimately impacting the emergence of trap states in perovskite films. Therefore, the composition of wide bandgap perovskite is important to increase the film quality and device performance. To provide more efficient and targeted guidance for their further optimization, it is crucial to investigate the complex relationship between the composition and the device performance of WBG PSCs [10,16].

The principal components of early WBG perovskite mainly focus on the MAPbI_3−x_Br_x_. Table 1 shows the performance statistics of MA-based wide bandgap perovskite solar cells. MAPbX perovskites have a stable photovoltaic phase in the operational window of photovoltaic devices [17]. In 2014, Huang et al. fabricated perovskite film MAPbI_3−x_Br_x_ with a band gap of 1.72 eV via the two-step preparation method, in which an isopropanol solution with mixed MABr and MAI was spin-coated onto the PbI_2_ layer, followed by a solvent annealing process [18]. In 2015, Hu et al. achieved 16.6% efficiency for MAPbBr_0.5_I_2.5_ perovskite devices by increasing grain size and crystallinity [19]. Additionally, adding Cs is essential to enhancing the open-circuit voltage, power conversion efficiency, and stability of state-of-the-art cells. Xie et al. overcame the problem of different initial particle aggregation affinity by introducing SCN^−^ and prepared a pure phase homogeneous MA_0.9_Cs_0.1_PbI_2_Br(SCN)_0.08_ perovskite film with increased grain size based on adding Cs [20].

**Figure 3:**
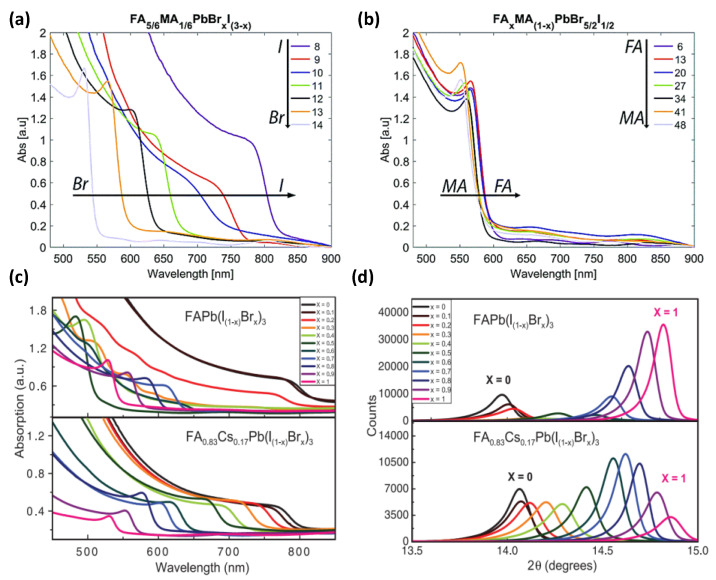
Optical absorption as a function of wavelength corresponding to (**a**) FA_5/6_MA_1/6_PbBr_x_I_3−x_ as the I/Br ratio is changed and (**b**) FA_x_MA_1−x_PbBr_5/2_I_1/2_ as the MA/FA ratio is changed [27]. Copyright © 2016, The Royal Society of Chemistry. (**c**) Ultraviolet–visible absorbance spectra of films of FAPb[I_(1−x)_Br_x_]_3_ and FA_0.83_Cs_0.17_Pb[I_(1−x)_Br_x_]_3_. a.u., arbitrary units. (**d**) XRD pattern of FAPb[I_(1−x)_Br_x_]_3_ and FA_0.83_Cs_0.17_Pb[I_(1−x)_Br_x_]_3_ [41]. Copyright © 2016, American Association for the Advancement of Science.

However, methylammonium (MA)-based perovskites have proven to be susceptible to environmental factors such as humidity, light, heat, and oxygen, all of which hurt the long-term operational stability of the device, thereby limiting its application in tandem solar cells. Formamidinium (FA)-based perovskites showed more excellent thermal stability than MA-based perovskites [24]. Table 2 shows the performance statistics of MA- and FA-based wide bandgap perovskite solar cells. Since perovskite (FAPbI_3_)_0.85_(MAPbBr_3_)_0.15_ was first reported, FA-based perovskites have been widely investigated due to their superior efficiency and improved stability [25]. Kim et al. found that the inclusion of formidine iodide (FAI) in (MAPbI_3_)_1−x_(MAPbBr_3_)_x_ could reduce the associated lattice strain and promote uniform perovskite films, resulting in an optimum efficiency of 18.68% [26]. Figure 3a,b shows the absorption spectra of FA_5/6_MA_1/6_PbBr_x_I_3−x_ and FA_x_MA_1−x_PbBr_2.5_I_0.5_ films as a function of wavelength [27]. Moreover, partially substituting FA with Cs can eliminate the phase instability region in the I to Br composition range. Consequently, mixed-cation hybrid halide perovskites (FA_x_MA_1−x_PbI_y_Br_3−y_) are widely investigated due to their tunable bandgap and excellent thermal stability.

**Table 2:** Summary of MA- and FA-based perovskite TSCs.

Absorber	E_g_ (eV)	V_oc_ (V)	J_sc_ (mAcm^−2^)	FF (%)	PCE (%)	Year	Ref
(FAPbI_3_)_0.8_(MAPbBr_3_)_0.2_	1.67	1.14	21.15	77.49	18.68	2019	[26]
FA_0.65_MA_0.20_Cs_0.15_Pb(I_0.8_Br_0.2_)_3_	1.68	1.17	21.2	79.8	19.8	2019	[28]
Cs_0.05_(FA_0.85_MA_0.15_)_0.95_Pb(I_0.85_Br_0.15_)_3_	1.62	1.135	22.8	78	20.25	2019	[29]
MA_0.9_FA_0.1_Pb(I_0.6_Br_0.4_)_3_	1.81	1.21	17.8	79.5	17.1	2020	[10]
Cs_0.05_FA_0.79_MA_0.16_Pb(I_0.6_Br_0.4_)_3_	1.75	1.26	19.19	76	18.38	2020	[30]
FA_0.65_MA_0.20_Cs_0.15_Pb(I_0.8_Br_0.2_)_3_	1.68	1.20	/	/	20.7	2020	[31]
Cs_0.05_(FA_0.77_MA_0.23_)_0.95_Pb(I_0.77_Br_0.23_)_3_	1.68	1.22	20.7	82.0	20.8	2020	[32]
FA_0.64_MA_0.20_Cs_0.15_Pb_0.99_(I_0.79_Br_0.2_)_3_	1.68	1.196	21.65	81.5	21.10	2020	[33]
FA_0.75_MA_0.15_Cs_0.1_Rb_0.05_PbI_2_Br	1.72	1.28	18.9	78.8	19.1	2021	[34]
Cs_0.15_MA_0.15_FA_0.7_Pb(I_0.8_Br_0.2_)_3_	1.68	1.22	/	/	20.5	2021	[35]
FA_0.75_MA_0.15_Cs_0.1_PbI_2_Br	1.74	1.19	18.69	78.21	17.32	2022	[36]
MA_0.96_FA_0.1_PbI_2_Br(SCN)_0.12_	1.72	1.19	18.65	78.4	17.40	2022	[37]
Cs_0.1_FA_0.2_MA_0.7_Pb(I_0.85_Br_0.15_)_3_	1.65	1.23	21.2	83.8	21.90	2022	[38]
FA_0.8_Cs_0.15_MA_0.05_Pb(I_0.82_Br_0.18_)_3_	1.65	1.221	21.5	83.3	21.90	2022	[39]
FAMACsPb(I_0.7_Br_0.3_)_3_	1.73	1.3	19.68	83.27	21.33	2023	[40]
FAMACsPb(I_0.6_Br_0.4_)_3_	1.79	1.34	17.80	83.10	19.53	2023	[40]
FAMACsPb(I_0.5_Br_0.5_)_3_	1.85	1.36	16.21	83.21	18.14	2023	[40]
FAMACsPb(I_0.4_Br_0.6_)_3_	1.92	1.39	14.30	83.47	16.23	2023	[40]

To overcome the instability of mixed I/Br WBG perovskites, many efforts focus on adjusting the composition of WBG perovskites. McMeekin et al. addressed the issues arising from low carrier mobility, high energy disorder, poor light-soaking stability, and thermal stability caused by phase segregation in WBG perovskite materials [41]. They fabricated a series of perovskite films composed of FAPb(I_1−x_Br_x_)_3_ (0 < x < 1) with mixed-cation FA_0.83_Cs_0.17_Pb(I_1−x_Br_x_)_3_ (0 < x < 1) for different Br contents. Notably, the lattice constant and bandgap variations conform perfectly to Vegard’s law within this range, as shown in Figure 3c,d. Table 3 shows the performance statistics of cesium–formamidinium (CsFA)-based wide bandgap perovskite solar cells. CsFA-based perovskites are preferable light-absorption materials due to their better stability. Ou et al. employed Cs_0.2_FA_0.8_Pb(I_0.8_Br_0.2_)_3_ MA-free perovskite as the light absorber for improving the stability of PSCs [42]. They further used interface modification and surface passivation to suppress the loss of interface transport barriers and nonradiative recombination, achieving a PCE of 20.11% and maintaining excellent stability.

## 3. Additive Engineering

Additive engineering has been considered a straightforward and effective strategy to further increase the perovskite film quality, decrease the trap density, and boost the general device efficiency [58,59,60]. For instance, adding a small quantity of organic cations to 3D perovskites can facilitate the formation of 2D and quasi-2D phases on the grain surface. This approach can concurrently eradicate the defects and enhance the stability of PSCs [48]. Kim et al. used two complementary additives (PEAI and Pb(SCN)_2_) to form 3D perovskite structures embedded in 2D (or quasi-2D) materials in a precursor solution, leading to enhanced perovskite morphology and reduced PbI_2_ formation, along with reduced defect density and energetic disorder [28].

**Figure 4:**
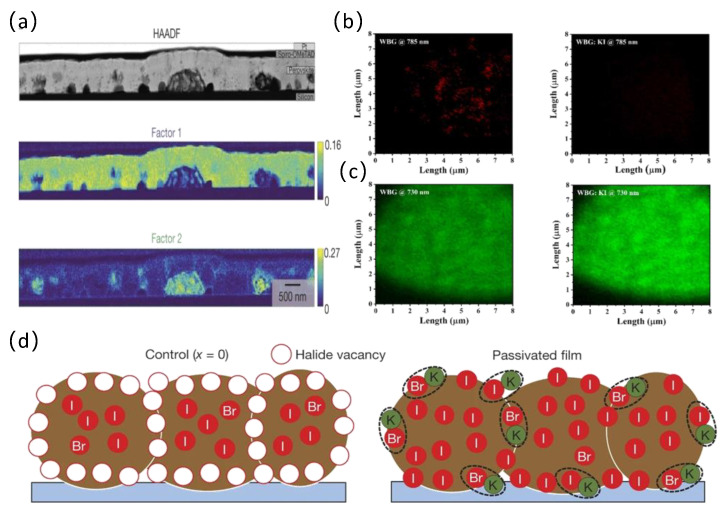
(**a**) HAADF STEM cross-sectional image of a (Cs, FA, MA)Pb(I_0.85_Br_0.15_)_3_ passivated perovskite thin film (x = 0.20) and analysis of STEM data using NMF decomposition reveals the presence of factor 1, associated with the perovskite layer, and of factor 2 [64]. Copyright © 2018, Springer Nature. (**b**) PL images of pristine and potassium-passivated WBG perovskite films at regions of 785 ± 5 nm (red), corresponding to peak energy around 1.57 eV. (**c**) PL imaging of pristine and potassium-passivated WBG perovskite films at regions of 730 ± 5 nm (green), corresponding to peak energy around 1.70 eV [45]. Copyright © 2020, American Chemical Society. (**d**) Schematic of a film cross-section showing halide-vacancy management in cases of excess halide, in which the surplus halide is immobilized through complexing with potassium into benign compounds at the grain boundaries and surfaces [64]. Copyright © 2018, Springer Nature.

In addition, various additives, such as Lewis acids, Lewis bases, low-dimensional perovskite, and ionic liquids, have been employed in wide bandgap perovskite solar cells. Prior research has confirmed that potassium is particularly beneficial in decreasing hysteresis and improving light stability for mixed-halide WBG perovskites [61,62,63]. Abdi-Jalebi et al. demonstrated that potassium could suppress the photoinduced ion migration in perovskite films and interfaces originating from the halide–potassium solid bonding energy (Figure 4a,d) [64]. Liang et al. demonstrated that potassium inactivation can significantly inhibit photoinduced phase segregation in mixed halide WBG perovskites, which could be verified by confocal PL microscopy imaging (Figure 4b,c) [45]. Furthermore, the introduction of KI could effectively reduce halide migration, improve crystal quality, and prolong carrier lifetime. By incorporating potassium hypophosphite into the perovskite precursor solution, Qiao et al. achieved an ultra-high V_oc_ of 1.32 V and a champion PCE of 20.06% by simultaneously adjusting the crystalline and passivating ion defects [65].

**Figure 5:**
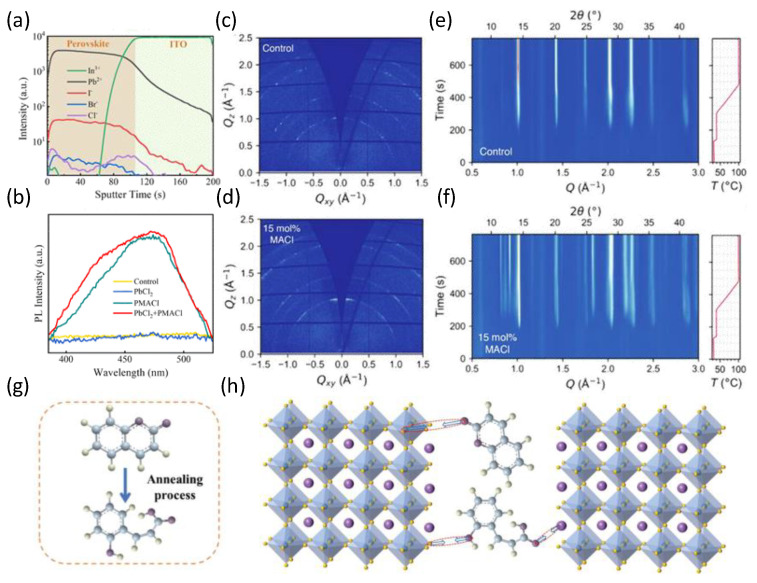
ToF-SIMS profiles of (**a**) the PbCl_2_ + PMACl perovskite film and PL spectra (**b**) of wide-Eg perovskite films with different additives [67]. Copyright © 2023, The Royal Society of Chemistry. (**c**,**d**) Two-dimensional GIWAXS patterns at 250 s into in situ thermal treatment for FA_0.83_Cs_0.17_Pb(I_0.6_Br_0.4_)_3_ without or with 15 mol% MACl additive [57]. (**e**,**f**) Time-resolved GIWAXS of FA_0.83_Cs_0.17_Pb(I_0.6_Br_0.4_)_3_ without or with 15 mol% MACl additives as the as-deposited materials crystallize during thermal treatment (temperatures are shown right) [57]. Copyright © 2023, John Wiley and Sons. (**g**) The thermal decomposition diagram of coumarin. (**h**) Schematic image of the interaction mechanism between perovskite and coumarin [69]. Copyright © 2023, John Wiley and Sons.

Several chlorine-containing additives, including ammonium chloride and methylammonium chloride, have also been utilized in precursors to improve the crystallinity of perovskites [66]. Zhao et al. developed a synergistic approach using lead chloride (PbCl_2_) and phenethylammonium chloride (PMACl) to introduce chlorine (Cl) into the bulk phase of the film, which could form a 2D phase on the surface of the perovskite film [67]. The incorporation of Cl into perovskite films could reduce the halide vacancies and hinder ion migration (Figure 5a). Additionally, the formation of a 2D perovskite phase induced by PMACl on grain surfaces could reduce nonradiative recombination and suppress phase segregation (Figure 5b). Subsequently, Shen et al. elucidated the crucial role of adding methyl ammonium chloride (MACl) in assisting the growth of the perovskite mesophase [57]. They concluded that the MACl additive can delay and regulate the crystallization of the WBG perovskite, as illustrated in Figure 5c–f. As a result, an open circuit voltage of 1.25 V and a power conversion efficiency of 17.0% were achieved for a 1.80 eV-based perovskite solar cell. However, metal halide perovskites unavoidably accumulate residual stresses during the annealing process, which precipitates undesirable defects and provokes phase segregation [68]. Recently, Xie et al. incorporated deformable coumarin into perovskite as a multifunctional additive to mitigate residual stress and suppress phase segregation [69]. The partial decomposition of coumarins during the annealing process could passivate perovskite film defects, resulting in prominent crystallinity and relatively large grain sizes by influencing the colloid size distribution, as shown in Figure 5g,h. Furthermore, Oliver et al. reduced nonradiative recombination within the perovskite film and interface transport layer by incorporating the ionic additive 1-butyl-1-methylpiperidinium tetrafluoroborate into the perovskite precursors [51]. Liu et al. employed potassium hexafluorophosphorus phosphate (KPF_6_) as a bifunctional additive to promote crystallization and reduce halide vacancies, resulting in an improved perovskite film and an excellent efficiency of 22.04% [70].

## 4. Interface Modification

The accumulated issues for carriers at the interface have still limited the efficiency of the layered perovskite solar cells. The interfacial defects between the perovskite and the CTL easily trap water and oxygen molecules, leading to the degradation of the perovskite film and the device’s long-term durability. In addition, interfacial defects usually increase the recombination of interfacial charge carriers and change the matching of interfacial energy levels, thereby affecting the performance and hysteresis of PSCs. The interface modification can effectively passivate the defects that existed at the interface of the CTL/perovskite layer, improve the quality of the perovskite film, and obtain matched energy level alignment between the CTL and perovskite layer, resulting in effective charge transfer and enhanced stability of perovskite solar cells. Therefore, interfacial modification is an effective way to reduce nonradiative recombination and improve the efficiency of PSCs.

**Figure 6:**
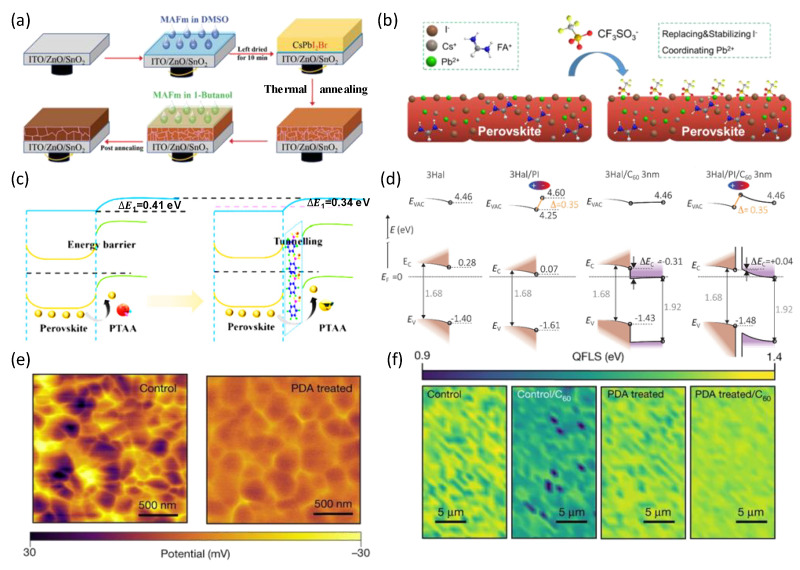
(**a**) Schematic diagram of the dual-interface engineering of perovskite and MAFm [76]. Copyright © 2023, John Wiley and Sons. (**b**) Schematic illustration of the CsCF_3_SO_3_ post-treatment [73]. Copyright © 2021, American Chemical Society. (**c**) Schematic representation of charge carrier transport with or without the addition of 5PFP-Br layer structure at the perovskite/PTAA interface [77]. Copyright © 2022, Elsevier. (**d**) Energy-level alignment of the 3Hal-PI and 3Hal-PI-C_60_ interfaces [78]. Copyright © 2023, The American Association for the Advancement of Science. (**e**) KPFM images of control and PDA-treated films. (**f**) QFLS maps of control and PDA-treated films demonstrating increased homogeneity and resistance to C_60_-induced defects [56]. Copyright © 2022, Springer Nature.

Several effective passivating agents have been employed for interface modification in PSCs, including small molecules [71], salt [72], zwitterions [73], polymers [74], and ionic liquids [75]. Recently, Sun et al. developed a dual-interface engineering approach based on the ionic liquid ammonium methyl formate (MAFm) to modify wide bandgap perovskite films, achieving a larger V_oc_ of 1.347 V [76]. The dual interface engineering could effectively promote uniform perovskite films with increased grain size, thereby reducing carrier recombination induced by defects and grain boundaries, as shown in Figure 6a. Chen et al. employed a post-treatment strategy with cesium sulfonate to attain an efficiency of 22.06% (Figure 6b), mainly attributed to the surplus iodide ions on the film surface, which decreased typical surface flaws [73]. Kang et al. introduced a 5-PFP-Br layer at the perovskite/PTAA interface, which passivated Pb defects and reduced grain boundaries in the perovskite films, resulting in increased carrier transfer and improved collection efficiency, as shown in Figure 6c [77]. The device performance was ultimately refined and improved via 5PFP-Br double interface passivation, resulting in an increased efficiency from 18.09% to 21.15% and an increased FF from 73.30% to 80.04%. Combining trihalide perovskite with piperazine iodide (3Hal) interface modification, Mariotti et al. obtained a high Voc of 1.28 V with improved band alignment (Figure 6d), reduced nonradiative recombination losses, and enhanced charge extraction at electron-selective contacts [78]. Zhang et al. achieved better device stability and device performance by attaching a surface passivation strategy with acetylcholine (ACh) [72]. The strategy could alter the band-edge state by introducing additional electronic states near the valence band maximum, thereby significantly reducing Voc losses by preventing ion migration. Li et al. formed an in-situ interlayer [74]. They facilitated hole migration from the perovskite layer to the spiro-OMeTAD layer by incorporating a dithiophenyl π-conjugated polymer (PDTBDT-FBT) into the perovskite. Subsequently, Li et al. used a naphthylenediimide (NDI)-based conjugated polymer with 3,4-difluorothiophene (PTzNDI-2FT) as a multifunctional interfacial layer between the perovskite and Spir-OMeTAD [79]. This approach effectively inhibited charge recombination, thereby preventing the formation of conduction channels between the perovskite and hole transport layer interfaces. As a result, charge transport from the perovskite layer to the HTL was improved. Chen et al. chemically modified the perovskite surface with 1,3-propane diammonium iodide (PDA), reducing interfacial recombination and achieving a more uniform surface potential distribution, as shown in Figure 6e,f [56]. The modified perovskite solar cells obtained a certified high voltage of 1.33 V and an efficiency of 19% with a bandgap of 1.79 eV. Zhao et al. used 1, 10-phenanthroline 5-amine (PAA) to tri-tooth anchor perovskite to reduce the injection barrier of interfacial charges, which could promote the extraction of interfacial holes and achieve a stable power conversion efficiency of 22.14% with a voltage of 1.207 V [71]. In addition, PAA passivation could inhibit the degradation of the perovskite film and enhance the water stability of perovskite solar cells. Liu et al. sequentially deposited ethylenediamine diiodide (EDAI_2_) and 4-fluorophenethyl ammonium chloride (4F-PEACl) on perovskite films through sequential interface engineering, achieving a higher PCE of 21.8% with an impressive V_oc_ of 1.262 V [80].

## 5. Wide Bandgap Perovskites/Silicon Tandem

Wide bandgap perovskite materials are excellent absorber materials to achieve high efficiency and current alignment with their suitable bandgap range of 1.68–1.72 eV. This makes perovskites uniquely suitable for tandem applications. Tandem solar cells based on perovskites combined with silicon have been considered the most ideal candidates in the photovoltaic energy field. Here, we mainly review the progress of 2T perovskite/silicon tandem technology due to its scientific complexity and potential practical advantages. Table 4 shows performance statistics for perovskite/silicon tandem solar cells. The rapid development of perovskite/silicon tandem solar cells is due to the optimization of perovskite components, the design of tandem structures, and the improvement of interface engineering. In addition, when designing 2T perovskite/silicon tandem devices, it is crucial to consider critical components, such as the top perovskite cell with a wide bandgap, the ICL, and the bottom silicon cell.

**Figure 7:**
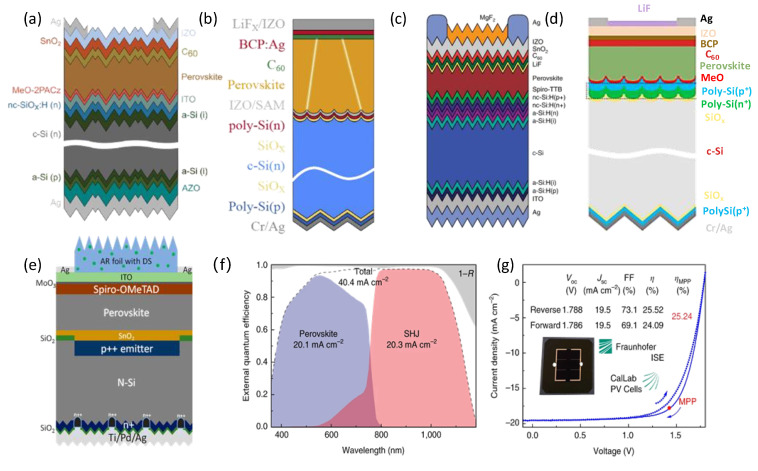
(**a**) Structure of a perovskite/SHJ tandem cell with an ITO recombination layer [108]. Copyright © 2021, John Wiley and Sons. (**b**) Structure of a perovskite/TOPCon tandem cell with an IZO recombination layer [96]. Copyright © 2022, Elsevier. (**c**) Structure of a perovskite/SHJ tandem cell with an nc-Si: H recombination layer [109]. Copyright © 2018, Springer Nature. (**d**) Structure of a 2T perovskite/TOPCon tandem cell with a doped poly-Si recombination layer [105]. Copyright © 2023, Springer Nature. (**e**) Structure of a perovskite/PERC tandem cell with RL-free [83]. Copyright © 2019 American Chemical Society. (**f**) EQE spectra of a current-matched fully textured monolithic perovskite/SHJ tandem cell and (**g**) Corresponding certified J–V data (1.42 cm^2^ aperture area) [109]. Copyright © 2018, Springer Nature.

The connection between the top and bottom cells is crucial for achieving high performance in a tandem cell. The interconnecting layer serves as an electrical connection and a transparent window layer for the bottom subcells. An ideal interconnecting layer should facilitate ohmic contact for efficient charge extraction and have enough recombination sites for the charges extracted from each subcell. Moreover, to prevent unwanted absorption within the ICL, it is imperative that it remain transparent to near-infrared light. The composition typically includes a transparent conductive oxide electrode (TCO) and a tunnel composite layer (TRL). In the absence of the TRL, the TCO-based rear-layer-free tandem solar cell relies on two transport layers with opposite polarities in the top and bottom cells to establish a p-n junction for carrier extraction and recombination; this configuration tends to exhibit lower efficiency levels, as shown in Figure 7.

Transparent conductive oxides, such as indium tin oxide (ITO) [101], zinc indium oxide (IZO) [96], and aluminum-doped zinc oxide (AZO), have been extensively employed as intermediate materials for interconnection layers (shown in Figure 7a,b). ITO is widely used as the interconnection layer for highly efficient perovskite/silicon tandem cells. ITO can be sputtered onto various substrates, possessing exceptional electrical conductivity and light transmission. However, the isotropic conductivity can lead to a mismatch between the refractive index of the ITO and the silicon substrate, resulting in a loss of light reflection in bands above 800 nm. Heavily doped trans-silicon-based materials, including a-Si: H and c-Si: H [109,110], have low transverse conductivity, parasitic loss, and reflection loss, making these materials ideal for a tunneling recombination layer. Sahli et al. prepared an n^+^/p^+^ nc-Si: H structure using plasma-enhanced chemical vapor deposition (PECVD) below 200 °C to replace ITO as the tunneling layer (Figure 7c), achieving a certified PCE of 25.2% [109]. This work provides an effective method to improve the optical loss at planar interconnecting interfaces. In addition, the polycrystalline silicon (poly-Si) stack consisting of poly-Si(p)/poly-Si(n) based on the tunnel oxide passivation contact (TOPCon) bottom subcells can provide excellent passivation and contact performance; therefore, it also has great application potential in perovskite/c-Si tandem solar cells [105,111,112].

**Figure 8:**
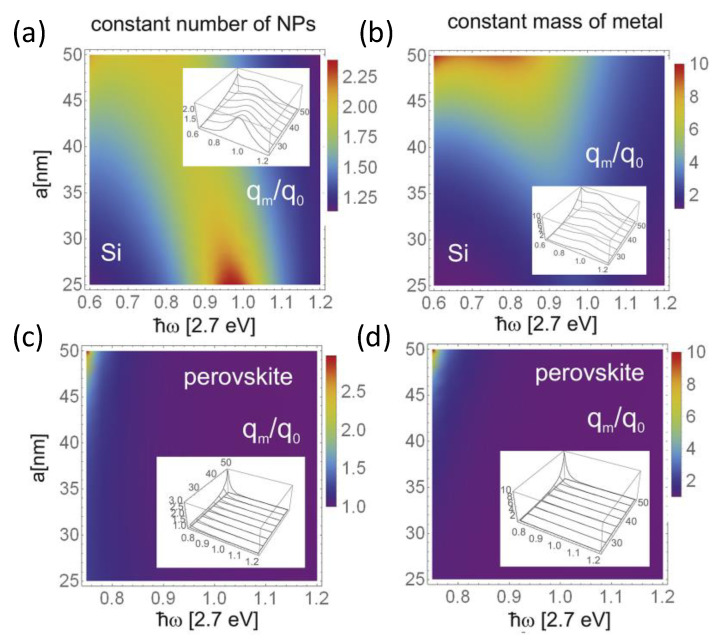
Spectral presentation of the efficiency increase q_m_/q_0_ for Si (**a**,**b**) and perovskite (**c**,**d**) cells covered with bare Au NPs. Left panels correspond to the kept-constant concentration of NPs when their size varies, whereas the right panels correspond to the total mass of all metallic components kept constant [113]. Copyright © 2020, Elsevier.

**Figure 9:**
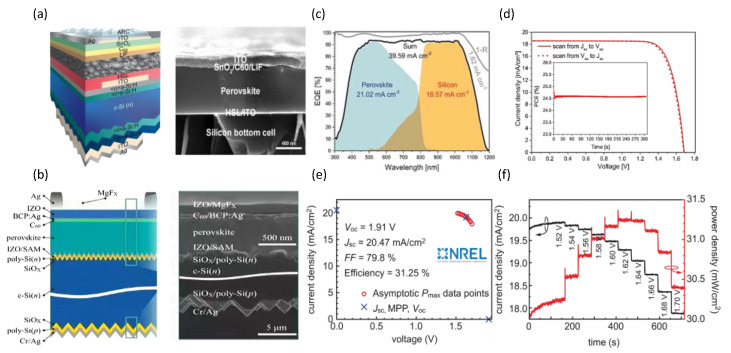
(**a**) Schematic diagram and cross-sectional scanning electron microscopy (SEM) image of a flat-structured silicon perovskite tandem solar cell [106]. Copyright © 2023, Elsevier. (**b**) Schematic diagram and cross-sectional scanning electron microscopy (SEM) image of a texture-structured silicon perovskite tandem solar cell [103]. Copyright © 2023, John Wiley and Sons. (**c**) EQE and reflection spectra (denoted as 1 − R), including the integrated current densities of the two subcells, the sum and reflection, and (**d**) J–V characteristics of a fully textured monolithic perovskite/silicon tandem solar cell with maximum power point tracking in the inset [108]. Copyright © 2021 John Wiley and Sons. (**e**) Asymptotic maximum power (Pmax) scan. (**f**) Current density and power density tracking around Pmax [104]. Copyright © 2023, The American Association for the Advancement of Science.

Different interconnect layers are tailored to other silicon substrates. The silicon bottom cells are crucial for achieving high efficiency in 2T tandem solar cells and maintaining the continuity of perovskite films. For complete Si-based solar cells, absorption strengthening is only the first element in a long series of factors resulting in improved efficiency. M Laska et al. further compared the absorption strengthening in Si and perovskites by similar application with metallization [113]. They found that the perovskites showed a poorer effect than Si solar cells, as shown in Figure 8. Therefore, the type and modification of Si solar cells are crucial for 2T perovskite/Si tandem solar cells. Crystalline silicon cells exhibited mature technology and stable electrical performance, further improving tandem solar cell efficiency. However, the type of crystalline silicon cells directly depends on the efficiency of perovskite/silicon tandem solar cells. According to the structural design, the silicon subcells can be classified as an aluminum back surface field (Al-BSF) cell, a silicon heterojunction (SHJ) cell (Figure 7c), a TOPCon cell (Figure 7d), a passivated emitter, and a rear contact (PERC) cell (Figure 7e). Furthermore, the morphology of the crystalline silicon cell exhibited a typical flat structure and texture structure, as illustrated in Figure 9a,b. Kim et al. fabricated perovskite/Al-BSF silicon tandem cells and achieved a power conversion efficiency of 21.19% with the optimized perovskite composition (FAPbI_3_)_0.8_(MAPbBr_3_)_0.2_ [26]. Zheng et al. utilized micron Eu^2+^ active phosphor as a bottom layer to fabricate perovskite/PERC tandem solar cells, attaining an efficiency of 23.1% [83]. Meanwhile, Yang’s team proposed a perovskite/TOPcon tandem cell with a certified efficiency of 28.76%, enabling efficient carrier transport and extraction via a tunnel composite layer of boron and phosphorus-doped polysilicon stacks [105]. The research team from Helmholtz Center in Berlin, Germany, achieved an efficiency of 24.6% based on the vaporized formamidine perovskite/textured SHJ tandem solar cell [108]. Additionally, the tandem solar cell showed excellent stability after 1000 h of light operation (Figure 9c,d). Moreover, the research team from the Swiss Federal Institute of Technology [109] in Lausanne developed a perovskite/SHJ tandem solar cell with a certified efficiency of 31.25% (Figure 9e,f).

## 6. Conclusions and Outlook

Our work reviews the recent advancements of wide bandgap perovskites towards 2T perovskite/silicon tandem solar cells. It emphasizes the effects of component engineering, additive engineering, and interface modification on wide bandgap perovskite solar cells and the application of perovskite/silicon tandem solar cells. Recently, the efficiency of perovskite/silicon tandem cells has steadily increased, culminating in a world record efficiency of 33.9%. Nevertheless, the power conversion efficiency of tandem solar cells still has considerable room for improvement compared with the theoretical efficiency. Novel technologies are required to prepare high-quality and micron-thick perovskite films suitable for large-scale manufacturing and match the texture of current silicon cells. The large area of perovskite film will be a critical factor in developing perovskite/Si tandem solar cells. Typed fabricated processes, such as blade-coating and slot die coating, have been widely used in upscalable perovskite films.

Furthermore, the stability, scalability, and cost-effectiveness of perovskite solar cells are several key issues to promote perovskite commercialization. Replacing MA with FA and introducing inorganic cations such as cesium and rubidium can further improve the phase and thermal stability of wide bandgap perovskite film. It is crucial to avoid the diffusion of metal ions and the reaction between the charge transport layer and the electrode by introducing a buffer layer [114]. Therefore, it is necessary to improve the stability and long-term performance of 2T perovskite/silicon tandem solar cells by optimizing the material composition, interface engineering, and packaging technology. In addition, material toxicity and life cycle assessment need to be further considered. In the life cycle stage, the environmental impact of raw material extraction, module manufacturing, organic solvents, and wastewater treatment in the module manufacturing process is significant. However, over a 20-year operating life, the potential toxicity of lead PSC generation will be ≈20 times lower than grid electricity [115]. When considering the different perovskite solar cells producing 1 kWh of electricity, the environmental impact depends largely on the conversion efficiency. Therefore, improved efficiency, reduced manufacturing costs, and component recycling programs may make PSCs very promising as a commercial PV technology.

While there are challenges such as those described above, more promising strategies are expected to overcome them. With the continuous breakthrough and improvement of technology, it is believed that it will play an essential role in the field of solar energy and contribute to the development of clean energy.

## Figures and Tables

**Table 1:** Summary of MA-based wide bandgap perovskite solar cells.

Absorber	E_g_ (eV)	V_oc_ (V)	J_sc_ (mAcm^−2^)	FF (%)	PCE (%)	Year	Ref
MAPbI_2.4_Br_0.6_	1.72	1.04	17.5	71.9	13.1	2014	[18]
MAPbI_2.5_Br_0.5_	1.70	1.16	18.3	78.2	16.6	2015	[19]
MA_0.9_Cs_0.1_PbI_2_Br(SCN)_0.08_	1.77	1.15	17.4	81.4	16.3	2019	[20]
Cs_0.1_MA_0.9_Pb(I_0.9_Br_0.1_)_3_	1.65	1.167	21.0	80.0	20.1	2020	[21]
MAPb(I_0.75_Br_0.25_)_3_	1.68	1.20	/	/	18.05	2020	[22]
MAPb(I_0.75_Br_0.25_)_3_	~1.73	1.22	20.85	81.11	20.59	2022	[23]

**Table 3:** Summary of CsFA-based wide bandgap perovskite solar cells.

Absorber	E_g_ (eV)	V_oc_ (V)	J_sc_ (mAcm^−2^)	FF (%)	PCE (%)	Year	Ref
FA_0.8_Cs_0.2_Pb(I_0.7_Br_0.3_)_3_	1.74	1.204	19.84	78	18.51	2019	[43]
Cs_0.4_FA_0.6_PbI_1.95_Br_1.05_	1.78	1.23	16.5	78.9	16.0	2020	[44]
FA_0.8_Cs_0.2_Pb(I_0.7_Br_0.3_)_3_	1.71	1.185	19.6	79	18.3	2020	[45]
Cs_0.22_FA_0.78_PbI_2.55_Br_0.45_	1.67	1.217	20.18	83.16	20.42	2020	[46]
FA_0.8_Cs_0.2_PbI_1.8_Br_1.2_	1.77	1.23	17.0	79.8	16.7	2021	[47]
Cs_0.2_FA_0.8_Pb(I_0.82_Br_0.15_Cl_0.03_)_3_	1.66	1.23	20.79	82.28	21.05	2021	[48]
FA_0.8_Cs_0.2_Pb(I_0.8_Br_0.2_)_3_	1.68	1.19	20.94	81.8	20.31	2021	[49]
Cs_0.22_FA_0.78_PbI_2.55−x_Br_0.45_Cl	1.68	1.204	20.72	81.73	20.39	2021	[50]
FA_0.83_Cs_0.17_Pb(I_0.6_Br_0.4_)_3_	1.79	1.22	/	/	17	2022	[51]
Cs_0.22_FA_0.78_Pb(I_0.85_Br_0.15_)_3_	1.65	1.21	21.08	80.49	20.53	2022	[52]
FA_0.8_Cs_0.2_Pb(I_0.7_Br_0.3_)_3_	1.75	1.21	19.3	86.5	20.2	2022	[53]
Cs_0.25_FA_0.75_Pb(I_0.85_Br_0.15_)_3_	1.65	1.20	22.15	83.81	22.33	2022	[54]
Cs_0.22_FA_0.78_Pb(I_0.85_Br_0.15_)_3_	1.67	1.19	20.33	81.7	19.76	2022	[55]
FA_0.8_Cs_0.2_Pb(I_0.6_Br_0.4_)_3_	1.79	1.33	18.06	84.2	20.2	2023	[56]
FA_0.83_Cs_0.17_Pb(I_0.6_Br_0.4_)_3_-15mol% MACl	1.80	1.25	/	/	17.0	2023	[57]
Cs_0.2_FA_0.8_Pb(I_0.8_Br_0.2_)_3_	1.66	1.20	21.02	79.91	20.11	2023	[42]

**Table 4:** Summary of perovskite/silicon tandem solar cells.

Absorber	Si		Interconnection Layers	E_g_ (eV)	V_oc_ (V)	J_sc_ (mAcm^−2^)	FF (%)	PCE (%)	Year	Ref
	Type	Morph								
Cs_0.05_(FA_0.83_MA_0.17_)_0.95_Pb(I_1−x_Br_x_)_3_	SHJ	flat	ITO	1.63	1.79	19.02	74.3	25.2	2019	[81]
(FAMAPbI_3_)_0.8_(MAPbBr_3_)_0.2_	AI_BSF	flat	ITO	1.64	1.65	16.1	79.9	21.19	2019	[26]
Cs_0.17_FA_0.83_Pb(I_0.83_Br_0.17_)_3_	SHJ	flat	ITO	1.63	1.74	18.53	75	24.5	2019	[18]
Cs_0.05_(MA_0.17_FA_0.83_)Pb_1.1_(I_0.83_Br_0.17_)_3_	SHJ	flat	ITO	1.63	1.76	18.5	78.5	25.5	2019	[82]
FAMAPbI_3−x_Br_x_	PERC	flat	Si(p++)	1.61	1.73	16.5	81	23.1	2019	[83]
FA_0.75_Cs_0.25_Pb(I_0.8_Br_0.2_)_3_	SHJ	flat	ITO	1.7	1.84	15.2	77.3	21.6	2019	[84]
Cs_0.1_MA_0.9_Pb(I_0.9_Br_0.1_)_3_	SHJ	textured	ITO	/	1.82	19.2	75.3	26.2	2020	[21]
Cs_0.05_MA_0.15_FA_0.8_PbI_2.25_Br_0.75_	SHJ	flat	InOx	1.68	1.78	19.07	75.4	25.7	2020	[85]
(FA_0.65_MA_0.2_Cs_0.15_)Pb(I_0.8_Br_0.2_)_3_	SHJ	flat	ITO	~1.7	1.76	19.2	79.2	26.7	2020	[31]
FA_0.75_Cs_0.25_Pb(I_0.8_Br_0.2_)_3_	SHJ	textured	ITO	1.68	1.77	17.7	80.3	25.1	2020	[86]
Cs_0.05_(FA_0.77_MA_0.23_)_0.95_Pb(I_0.77_Br_0.23_)_3_	SHJ	textured	ITO	1.68	1.90	19.26	79.52	29.15	2020	[32]
Cs_0.1_MA_0.9_Pb(I_0.9_Br_0.1_)_3_	SHJ	textured	/	/	1.88	20.26	77.3	29.5	2020	[87]
Cs_0.05_(MA_0.23_FA_0.77_)_0.95_Pb(Br_0.23_I_0.77_)_3_	SHJ	flat	ITO	1.68	1.89	19.13	78.0	28.2	2021	[88]
Cs_0.05_(MA_0.23_FA_0.77_)_0.95_Pb(Br_0.23_I_0.77_)_3_	SHJ	flat	nc-SiOx(n) /ITO	1.68	1.94	17.81	80.9	27.9	2021	[88]
Cs_0.15_MA_0.15_FA_0.70_Pb(I_0.80_Br_0.20_)_3_	SHJ	textured	ITO	1.68	1.84	19.6	76.0	27.4	2021	[35]
Cs_0.05_MA_0.15_FA_0.8_Pb(I_0.75_Br_0.25_)_3_	SHJ	textured	ITO	1.68	1.83	19.5	75.9	27.1	2021	[89]
Cs_0.15_MA_0.15_FA_0.70_Pb(I_0.80_Br_0.20_)_3_	SHJ	flat	ITO	1.68	1.78	19.2	76.8	26.2	2021	[90]
FA_0.9_Cs_0.1_PbI_2.87_Br_0.13_	SHJ	textured	ITO	1.645	1.81	19.78	76.9	27.5	2021	[91]
Cs_0.05_FA_0.8_MA_0.15_Pb(I_0.75_Br_0.25_)_3_	SHJ	textured	ITO	1.65	1.88	19.1	75.5	27.1	2021	[92]
Cs_0.22_FA_0.78_Pb(I_0.85_Br_0.15_)_3_	SHJ	flat	a-Si:H /ITO	1.64	1.86	76.22	79.23	27.26	2022	[55]
Cs_0.1_FA_0.2_MA_0.7_Pb(I_0.85_Br_0.15_)_3_	SHJ	textured	a-Si:H /ITO	1.65	1.92	18.95	78.5	28.56	2022	[38]
Cs_0.22_FA_0.78_Pb(Cl_0.03_Br_0.15_I_0.85_)_3_	TOPCon	flat	poly-TPD /ITO	1.68	1.79	19.68	78.27	27.63	2022	[93]
FA_0.78_Cs_0.22_Pb(I_0.85_Br_0.15_)_3_	PERC	flat	IZO	1.68	1.91	19.29	78.3	28.81	2022	[94]
Cs_x_FA_y_MA_1−(x+y)_Pb(I,Br)_3_	SHJ	textured	ITO	1.63	1.80	19.83	79.6	28.40	2022	[95]
Cs_0.05_(FA_0.83_MA_0.17_)Pb_1.1_(I_0.83_Br_0.17_)_3_	TOPCon	textured	poly-Si /IZO	1.63	1.80	19.3	81.9	28.5	2022	[96]
Cs_0.05_(FA_0.77_MA_0.23_)_0.95_Pb(I_0.77_Br_0.23_)_3_	SHJ	textured	ITO	1.68	1.92	19.48	79.4	29.75	2022	[97]
Cs_0.1_FA_0.9_PbI_2.74_Br_0.16_Cl_0.1_	SHJ	textured	nc-SiOx	1.63	1.85	19.35	79.62	28.51	2022	[98]
Cs_0.05_MA_0.14_FA_0.81_Pb(I_0.8_Br_0.2_)_3_	SHJ	textured	IZO	1.68	1.85	19.7	77.9	28.4	2022	[99]
Cs_0.05_(FA_0.83_MA_0.17_)_0.95_Pb(I_0.83_Br_0.17_)_3_	TOPCon	textured	poly-Si/ IZO	1.63	1.8	19.4	81.64	28.49	2022	[100]
Cs_0.05_(FA_0.83_MA_0.17_)_0.95_Pb(I_0.83_Br_0.17_)_3_	TOPCon	flat	poly-Si/ IZO	1.63	1.75	18.2	80.3	25.65	2022	[100]
(FAPbI_3_)_0.83_(MAPbBr_3_)_0.17_	SHJ	flat	ITO	/	1.82	18.1	82.4	27.2	2023	[101]
(CsI)_0.08_(PbI_1.4_Br_0.6_)	SHJ	flat	IZO	1.67	1.9	19.48	76.42	28.35	2023	[102]
Cs_0.05_(FA_0.83_MA_0.17_)_0.95_Pb(I_0.83_Br_0.17_)_3_	TOPCon	textured	IZO	1.63	1.85	19.4	81.8	29.3	2023	[103]
Cs_0.18_FA_0.82_Pb(I,Br)_3_	SHJ	textured	IZO	1.7	1.91	20.47	79.8	31.25	2023	[104]
Cs_0.05_(FA_0.83_MA_0.17_)_0.95_Pb(I_0.83_Br_0.17_)_3_	TOPCon	textured	IZO	/	1.83	19.7	81	29.2	2023	[105]
Cs_0.15_FA_0.65_MA_0.2_Pb(I_0.8_Br_0.2_)_3_	SHJ	textured	ITO	1.67	1.91	19.1	79.1	28.9	2023	[106]
Cs_0.05_FA_0.8_MA_0.15_Pb(I_0.75_Br_0.25_)_3_	SHJ	textured	IZO	1.68	1.849	20.1	77.6	28.8	2023	[107]

## Data Availability

Data are available upon request.
